# Hospitalization rates from radiotherapy complications in the United States

**DOI:** 10.1038/s41598-022-08491-8

**Published:** 2022-03-14

**Authors:** Raees Tonse, Venkataraghavan Ramamoorthy, Muni Rubens, Anshul Saxena, Peter McGranaghan, Emir Veledar, Matthew D. Hall, Michael D. Chuong, Manmeet S. Ahluwalia, Minesh P. Mehta, Rupesh Kotecha

**Affiliations:** 1 Department of Radiation Oncology, Miami Cancer Institute, Baptist Health South Florida, Office 1R203, Miami, FL 33176 USA; 2 Baptist Health South Florida, Miami, FL USA; 3 Office of Clinical Research, Miami Cancer Institute, Baptist Health South Florida, Miami, FL USA; 4 Herbert Wertheim College of Medicine, Florida International University, Miami, FL USA; 5 Department of Medical Oncology, Miami Cancer Institute, Baptist Health South Florida, Miami, FL USA

**Keywords:** Cancer, Oncology

## Abstract

Hospitalizations due to radiotherapy (RT) complications result in significant healthcare expenditures and adversely affect the quality of life of cancer patients. Using a nationally representative dataset, the objective of this study is to identify trends in the incidence of these hospitalizations, their causes, and the resulting financial burden. Data from the National Inpatient Sample was retrospectively analyzed from 2005 to 2016. RT complications were identified using ICD-9 and ICD-10 external cause-of-injury codes. The hospitalization rate was the primary endpoint, with cost and in-hospital death as secondary outcomes. 443,222,223 weighted hospitalizations occurred during the study period, of which 482,525 (0.11%) were attributed to RT. The 3 most common reasons for RT-related hospitalization were cystitis (4.8%, standard error [SE] = 0.09), gastroenteritis/colitis (3.7%, SE = 0.07), and esophagitis (3.5%, SE = 0.07). Aspiration pneumonitis (1.4-fold) and mucositis (1.3-fold) had the highest relative increases among these hospitalizations from 2005 to 2016, while esophagitis (0.58-fold) and disorders of the rectum and anus were the lowest (0.67-fold). The median length of stay of patient for hospitalization for RT complications was 4.1 (IQR, 2.2–7.5) days and the median charge per patient was $10,097 (IQR, 5755–18,891) and the total cost during the study period was $4.9 billion. Hospitalization for RT-related complications is relatively rare, but those that are admitted incur a substantial cost. Use of advanced RT techniques should be employed whenever possible to mitigate the risk of severe toxicity and therefore reduce the need to admit patients.

## Introduction

Cancer is one of the most common causes of morbidity and mortality in the United States, with an estimated 1,806,590 new cancer cases^1^. A substantial proportion of cancer patients with solid tumors undergo radiotherapy at some point during their disease course^2, 3^. Each year approximately 650,000 individuals in the United States receive radiotherapy or chemotherapy^4^. Although RT offers significant clinical benefit to cancer patients, either curative or palliative, adverse effects are possible some of which may be severe and ultimately require hospitalization^5, 6^. Hospitalizations among cancer patients undergoing RT and chemotherapy have been evaluated and reported in several studies^5, 7–11^. Majority of these studies report that neutropenia, thrombocytopenia, anemia and infections to be the leading causes of hospitalization. Jairam et al. evaluated the financial burden of treatment-related complications of systemic therapy and radiotherapy for patients who were treated in emergency departments in the United States. However, there was a paucity of data from a nationally representative dataset that showed the incidence and hospitalizations due to RT complications.

Hospitalization for RT complications entails high costs and is a huge burden on the patients and healthcare system. According to a MedStat analysis from 2007, each of these hospitalizations cost an average of $22,000 per patient^12^. Furthermore, hospitalization causes treatment disruptions and can adversely affect treatment response. Understanding the nature of these hospitalizations could aid in the development of preventive strategies to in the outpatient setting. Although similar studies have been performed for chemotherapy-related toxicities and hospitalizations, to our knowledge a comprehensive analysis of hospitalizations resulting from RT complications at the national level has not been done. Using a nationally representative dataset, we have aimed to classify the incidence and causes of these hospitalizations and the financial burden associated with these hospitalizations.

## Methods

### Data source

To characterize hospitalizations for RT complications, we analyzed data from the National Inpatient Sample (NIS) from 2005 to 2016. The NIS was created by the Agency for Healthcare Research and Quality (AHRQ) as part of the Healthcare Cost and Utilization Project (HCUP) and is the largest all-payer inpatient database in the United States^13^. Researchers and policymakers can utilize NIS to estimate factors including healthcare utilization, hospitalization costs and overall national healthcare costs, all of which are helpful in making healthcare policy decisions. Every year, the NIS collects data from over 7 million hospitalizations in the United States. amounting to 35 million weighted hospitalizations. In 2012, the NIS revised its data collection process in order to obtain a more representative sample of national estimates. Prior to 2012, the NIS gathered and collected a sample of discharge records from a small number of HCUP hospitals. Aside from demographics, hospital data, clinical procedures, length of stay, disposition status, and overall costs, each hospitalization datafile comprises one primary and up to 29 secondary diagnoses. Primary and secondary diagnoses were reported using *Classification of Diseases, Ninth Revision, Clinical Modification (ICD-9-CM)* codes and *Tenth Revision (ICD-10-CM)* codes.

### Study design

Cancer patients were identified in the NIS using Classifications Software (CCS) codes 11–45^14^. Cancers were divided into two categories: solid tumors and hematologic malignancies (see Table 1). External cause-of-injury codes (E-codes) from ICD-9-CM and ICD-10-CM were used to identify RT complications. The primary reason for hospitalization was the first reported non-cancer disease identified using an ICD-9-CM or ICD-10-CM diagnosis code (Supplemental Table 1). The CONSORT diagram depicts the technique for selecting the patient cohort (Supplemental Fig. 1). The study examined demographics such as age, gender, and race; socioeconomic factors included median household income by zip code, insurance type; and hospital characteristics included region, bed size, and teaching status. The key end measures were the hospitalization rate and total cost, while the length of stay in the hospital and in-hospital mortality for RT complications were secondary outcomes. Our findings were reported using the STROBE (Strengthening the Reporting of Observational Studies in Epidemiology) guidelines. The methods for this study were conducted in accordance with relevant guidelines and regulations. The study was reviewed by the Miami Cancer Institute’s Institutional Review Board, which exempted the study from institutional review board approval and waived the requirement for informed consent because it uses previously collected deidentified data stored in NIS.

**Table 1:** Hospitalizations for complications of radiotherapy by tumor types, 2006–2015.

Tumor type	Number of hospitalizations, n (%)
**Solid**	
Lung	100,392 (20.8%, 95% CI 19.1–21.6%)
Head and neck	58,073 (12.0%, 95% CI 10.8–12.7%)
Rectum and anus	35,134 (7.3%, 95% CI 6.8–8.1%)
Breast	43,345 (9.0%, 95% CI 8.2–9.9%)
Cervix	28,162 (5.8%, 95% CI 4.8–6.7%)
Uterus	17,918 (3.7%, 95% CI 3.1–4.4%)
Colon	17,505 (3.6%, 95% CI 3.2–4.4%)
Bladder	14,951 (3.1%, 95% CI 2.6–3.8%)
Esophagus	13,318 (2.8%, 95% CI 2.1–3.5%)
Other	11,546 (2.4%, 95% CI 1.8–3.2%)
Brain and nervous system	10,922 (2.3%, 95% CI 1.8–3.1%)
Bone and connective tissue	8474 (1.8%, 95% CI 0.9–2.7%)
Prostate	83,771 (17.4%, 95% CI 16.8–18.1%)
Kidney and renal	6391 (1.3%, 95% CI 0.7–2.0%)
Stomach	5704 (1.2%, 95% CI 0.8–1.9%)
Ovary	5357 (1.1%, 95% CI 0.7–1.9%)
Melanoma	5108 (1.1%, 95% CI 0.7–1.8%)
Thyroid	4845 (1.0%, 95% CI 0.6–2.0%)
Pancreas	4506 (0.93%, 95% CI 0.67–1.35%)
Liver and intrahepatic bile duct	2579 (0.53%, 95% CI 0.32–0.98%)
Testis	1375 (0.29%, 95% CI 0.09–0.42%)
**Liquid**	
Non-Hodgkin lymphoma	16,842 (3.5%, 95% CI 3.2–4.4%)
Hodgkin lymphoma	6814 (1.4%, 95% CI 0.7–2.0%)
Leukemia	6698 (1.4%, 95% CI 0.8–1.9%)
Multiple myeloma	3787 (0.78%, 95% CI 0.57–1.2%)

### Statistical analysis

SAS (version 9.4, SAS Institute, Cary, North Carolina) was used to do statistical analysis. The study followed the guidelines provided by Khera and Krumholz for using NIS data^15^. The NIS was updated in 2012 to be more nationally representative, as previously noted. We used modified discharge weights for the years 2005–2011 to account for these changes in data collection^16^. Temporal factors, cancer types, demographics, socioeconomic factors, and hospital characteristics were all evaluated using descriptive statistics. Frequencies and percentages were used to describe categorical variables and median and interquartile ranges were used for continuous variables. The total number of hospitalizations due to RT complications was divided by the total number of hospitalizations to calculate the hospitalization rates. Total hospital charges and cost-to-charge ratios were multiplied to calculate the costs of individual inpatient stays. The expenditures for each year were adjusted based on 2016 inflation levels using the US Consumer Price Index. By subtracting the admission date from the discharge date, the length of stay in the hospital was calculated. The number of patients admitted for RT complications who died in the hospital was divided by the total number of patients hospitalized for RT complications to calculate the In-hospital mortality. Weighted frequencies were used in all analyses to calculate national estimates using discharge weight, or "DISCWT."

### Ethics approval statement

Since the study uses previously gathered deidentified data maintained in NIS, the study was excused from the institutional review board approval. All of the methodologies followed the appropriate ethical requirements for handling data.

### Informed consent statement

Since this study involves an administrative database that does not contain any personal information that may be connected to an individual participant, informed consent was not essential.

## Results

### Characteristics of the cohort

Between 2005 and 2016, 443,222,223 weighted hospitalizations were documented, with 482,525 of them due to RT complications. Median age of the patients was 66.4 (interquartile range [IQR, 56.0–76.0]) years. More than half of the patients were ≥ 65 years old. The male-to-female distribution was relatively equal (52.8 vs. 47.2%). Majority of these patients were white (70.5%, 95% CI 69.1–71.2%)), followed by blacks (9.8%, 95% CI 8.1–10.7%) and Hispanics (5.8%, 95% CI 4.3–6.5%) (Supplemental Table 2). Almost two-thirds of patients were admitted to hospitals with large bed size (65.3%, 95% CI 64.3–66.2%) and urban teaching hospitals (57.9%, 95% CI 56.1–58.7%). The bulk of patients were admitted in the South (37.0%, 95% CI 35.7–38.2%), according to the region-wise distribution. The in-hospital mortality rate due to complications of RT was 3.6% (95% CI 2.4–4.6%). The average annual percentage change (AAPC) in hospitalization rates for radiation complications was 1.1% during the study period, compared to -0.5% for general hospitalizations.

**Figure 1:**
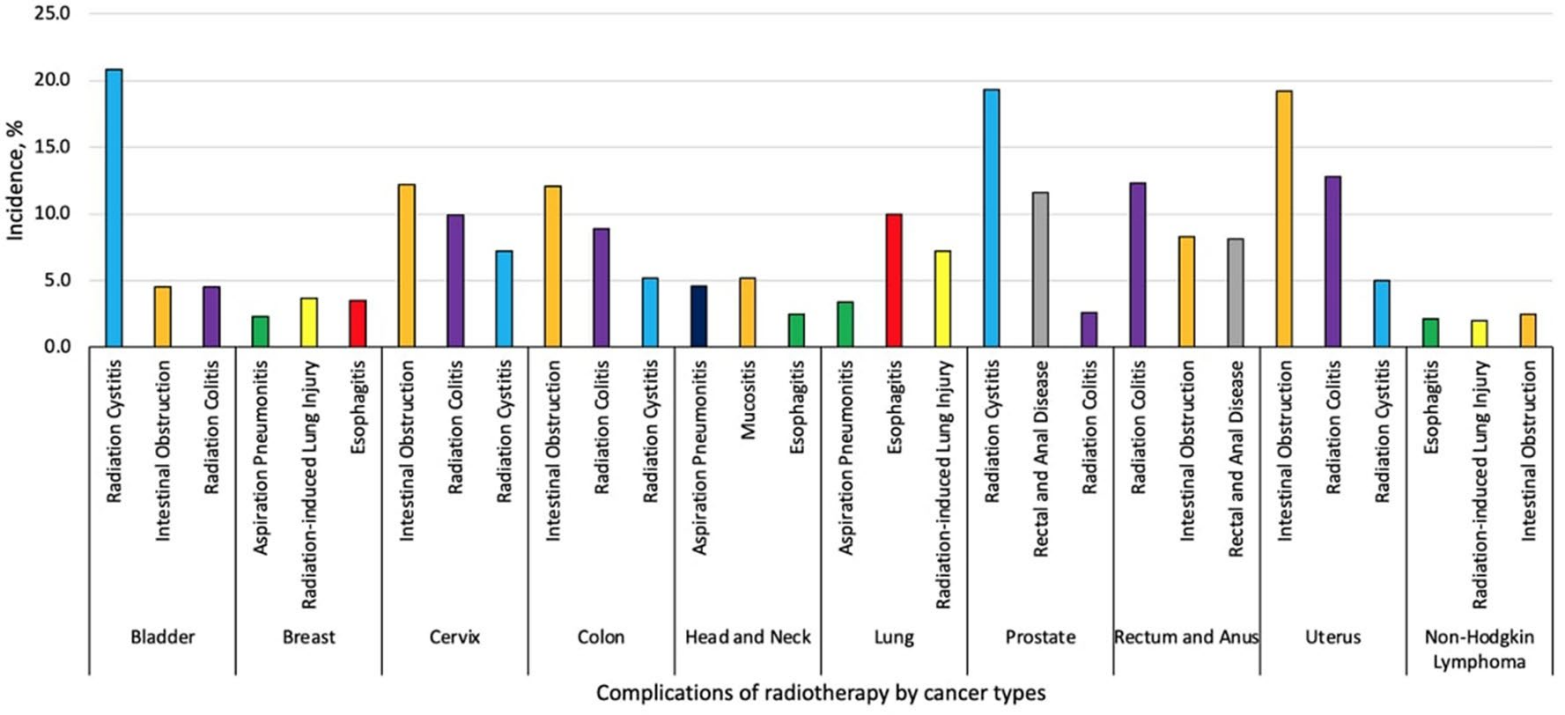
Percentage of patients admitted for RT related complications by most common cancer types.

We also analyzed radiotherapy complications by cancer type. Lung (20.8%, 95% CI 19.1–21.6%), prostate (17.4%, 95% CI 16.8–18.1%), and head and neck (12.0%, 95% CI 10.8–12.7%) cancers were the most common cancers leading to hospitalization. Among solid tumors, the most common complications were radiation cystitis (5.0%, 95% CI 4.1–5.6%)), intestinal obstruction (without hernia) (4.2%, 95% CI 3.6–5.1%)), and radiation-induced gastroenteritis and colitis (3.8%, 95% CI 3.1–4.4%), and among hematologic malignancies, the most common complications were intestinal obstruction (without hernia) (5.5%, 95% CI 4.8–6.7%), radiation-induced lung injury (5.1%, 95% CI 4.6–6.6%), and esophagitis (2.5%, 95% CI 1.8–3.2%). Figure 1 demonstrates the most common complications for the ten most common cancer types. Intestinal obstruction (without hernia) was among the top three complications for bladder, cervix, colon, rectum, anus, and uterine cancers as well as for non-Hodgkin lymphoma. Esophagitis was among the top three complications for breast, head and neck, lung cancers and non-Hodgkin lymphoma. Radiation-induced gastroenteritis and colitis was commonly seen in cancers of rectum and anus (24.6%, 95% CI 22.2–25.9%), uterus (13.0%, 95% CI 11.1–14.8%), prostate (11.4%, 95% CI 10.2–12.7%) and colon (8.8%, 95% CI 8.1–9.9%). Disorders of rectum and anus was seen in cancers of prostate (57.8%, 95% CI 56.1–58.9%), rectum and anus (16.8%, 95% CI 15.1–17.1%)), and cervix (8.8%, 95% CI 8.1–10.3%).

### Factors associated with inpatient admission and mortality

**Figure 2:**
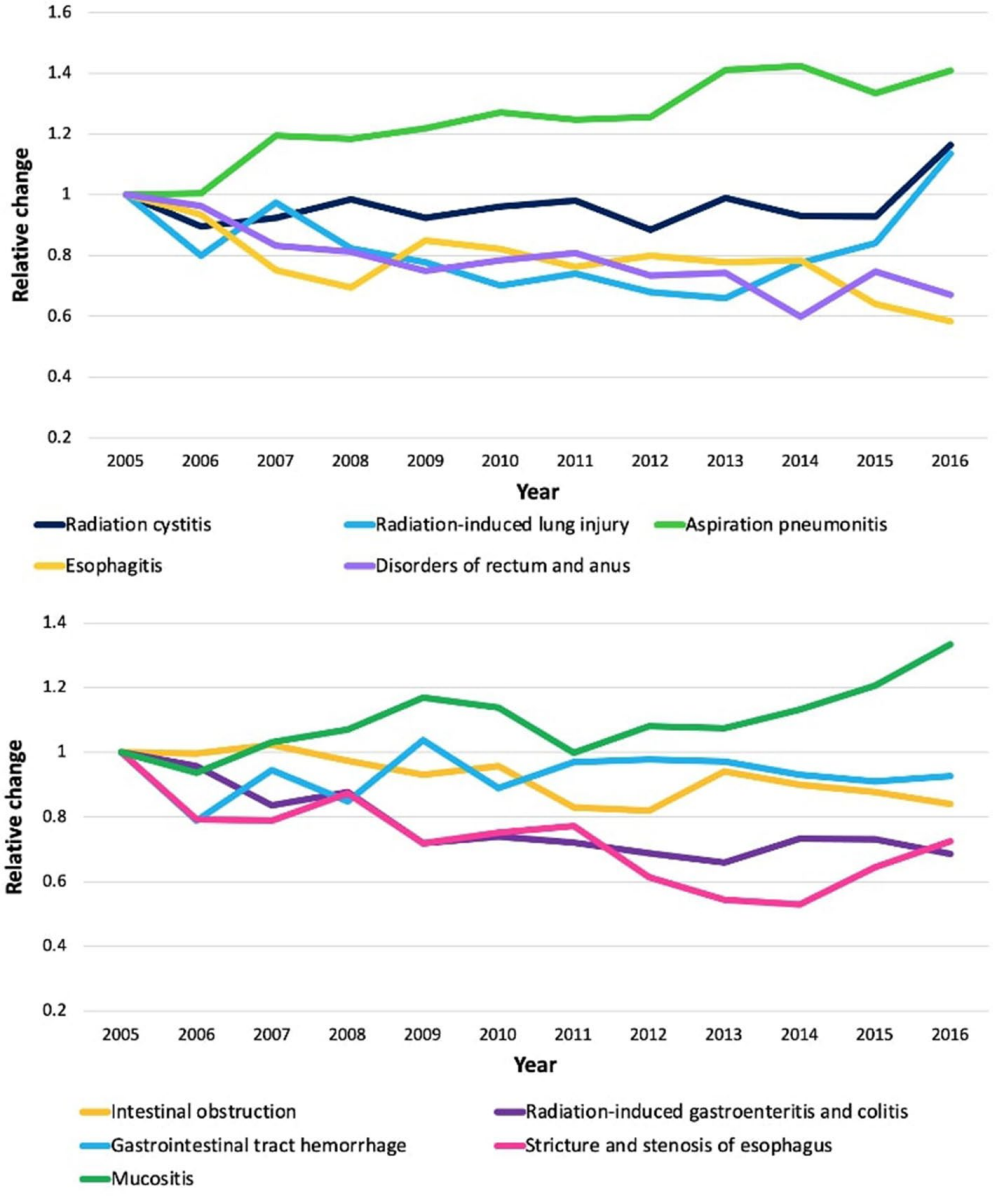
Relative change in number of hospitalizations for radiation cystitis, radiation-induced lung injury, aspiration pneumonitis, esophagitis, and disorders of rectum and anus, intestinal obstruction, radiation-induced gastroenteritis and colitis, gastrointestinal tract hemorrhage, stricture and stenosis of esophagus, and mucositis from 2006 to 2015.

Aspiration pneumonitis (1.4-fold) and mucositis (1.3-fold) had the highest relative increases among these hospitalizations from 2005 to 2016, while esophagitis (0.58-fold) and disorders of the rectum and anus were the lowest (0.67-fold) (see Fig. 2). Table 2 depicts the complications associated with RT with respect to the number of hospitalizations, duration of stay in the hospital, in-hospital mortality, and expenses. The top reasons for hospitalizations were radiation cystitis (4.8%, 95% CI 4.2–5.3%), radiation-induced gastroenteritis and colitis (3.7%, 95% CI 3.2–4.2%), and esophagitis (3.5%, 95% CI 3.1–3.9%). The length of stay was highest for aspiration pneumonitis (5.9 days, interquartile range [IQR] 3.3–10.5), intestinal obstruction without hernia (4.8 days, IQR 2.5–9.7), and mucositis (4.8 days, IQR 2.7–8.1). In-hospital mortality associated with RT were aspiration pneumonitis (9.6%, 95% CI 8.1–10.9%), radiation-induced lung injury (7.9%, 95% CI 6.1–8.9%), and stricture and stenosis of esophagus (3.4%, 95% CI 2.2–4.7%). Total hospitalization cost for non-RT was $3.2 trillion and for RT was $6.1 billion (0.20% of total hospitalization cost). The costliest complications were aspiration pneumonitis ($13,859), intestinal obstruction without hernia ($10,386), and mucositis ($9,947). The median length of stay of patient for hospitalization for RT complications was 4.1 (IQR, 2.2–7.5) days and the median charge per patient was $10,097 (IQR, 5755–18,891) and the total cost during the study period was $4,9 billion.

**Table 2:** Number of hospitalizations, hospital length of stay, in-hospital mortality, and charges for top 10 complications of radiotherapy, 2006–2015.

Diagnosis	Number of hospitalizations (%)	Length of stay in days, median (IQR)	Mortality, %	Charges per hospitalization in USD	Total charges during study period in USD
Radiation cystitis	23,161 (4.8%, 95% CI 4.2–5.3%)	3.6 (1.9–6.4)	1.1	8107	180,642,271
Radiation-induced gastroenteritis and colitis	17,853 (3.7%, 95% CI 3.2–4.2%)	4.1 (2.3–7.5)	1.3	8925	151,254,009
Esophagitis	16,888 (3.5%, 95% CI 3.1–3.9%)	4.4 (2.5–7.6)	1.5	8791	130,639,198
Disorders of rectum and anus	16,888 (3.5%, 95% CI 3.1–3.9%)	2.8 (1.6–4.8)	0.57	6675	102,610,160
Intestinal obstruction without hernia	16,888 (3.5%, 95% CI 3.1–3.9%)	4.8 (2.5–9.7)	1.4	10,386	191,125,253
Radiation-induced lung injury	10,133 (2.1%, 95% CI 1.8–2.6%)	4.3 (2.4–7.4)	7.9	9651	100,383,570
Gastrointestinal tract hemorrhage	9,658 (2.0%, 95% CI 1.7–2.5%)	2.9 (1.6–5.0)	2.9	7608	68,492,085
Aspiration pneumonitis	6,273 (1.3%, 95% CI 0.7–2.0%)	5.9 (3.3–10.5)	9.6	13,859	77,942,045
Stricture and stenosis of esophagus	4,735 (0.98%, 95% CI 0.77–1.25%)	4.0 (2.0–7.2)	3.4	9022	43,007,538
Mucositis	4,593 (0.95%, 95% CI 0.76–1.24%)	4.8 (2.7–8.1)	1.7	9947	36,977,567

## Discussion

To our knowledge, this is the first study to use a nationally representative dataset to report hospitalizations for RT complications. There was an overall decrease in hospitalizations between 2005 and 2016, but hospitalizations related to complications from RT remained stable over the same time period. A vast majority of these hospitalizations occurred in adults > 60 years of age. In terms of insurance providers and hospital locations associated with admission, Medicare and urban teaching centers were dominant. The complications associated with the highest frequency of hospitalization were radiation cystitis, radiation-induced gastroenteritis and colitis, and esophagitis.

The steady rate of hospitalizations for complications of RT, in spite of an overall increase in the number of patients receiving RT, is likely explained by the overall decrease in adverse events associated with RT over time because of technical improvements and better management of toxicities in the outpatient setting. Factors such as improved image guidance during treatment, advanced treatment planning systems and highly conformal and accurate delivery techniques of radiotherapy such as intensity-modulated radiation therapy (IMRT), image-guided radiation therapy (IGRT), volumetric-modulated arc therapy (VMAT), stereotactic radiotherapy and particle therapy (proton beam therapy) have been increasingly adopted in the last few years, and have reduced the dose delivered to normal structures thereby decreasing the intensity of radiation associated side effects^17^.

In this analysis, we noted that the most patients were admitted to large urban teaching hospitals. One possible explanation for this could be the fact that RT facility involves high investment and is usually only available at larger hospitals, and patients are prone to seeking care at hospitals where they received their RT^18^. By 2030, it is projected that 74.1 million (20.6%) Americans will be 65 years or older, and cancer incidence is expected to increase by 67% between 2010 and 2030^19^. A significant proportion of these patients will receive RT as part of their treatment^20^. In our study, we discovered that more than half of the patients who required hospitalization after RT were 65 years and above. Understanding the unique differences that older cancer patients have as compared to their younger counterparts, both in terms of tumor biology, overall health and functional status is critical in approaching the management in this patient population^21, 22^. The treating physicians may find it beneficial to have a working understanding of the available methods for completing a geriatric assessment that may affect treatment outcomes in older adults in order to provide comprehensive care^23^.

Radiation therapy is a localized treatment, therefore the complications that may arise are limited to the organs that are in close proximity to the tumor^24^. In our study, esophagitis was common among lung and breast cancers since esophagus is the one of the organs at risk (OAR) in these tumors. Symptoms such as throat pain, dysphagia, and the feeling that food is stuck in the chest normally appear 2 to 3 weeks after treatment^25^. The more the esophagus is included in the radiation field (the region of the body receiving radiation), the higher the risk of esophagitis. Furthermore, with the introduction of hypofractionation, the patient is more likely to develop esophagitis^26^. Similarly, intestinal obstruction was also found to be common among cancers of the bladder, cervix, colon, rectum, and uterus, owing to the close proximity of the bowels, which serve as the OAR for these tumors. This condition develops as a result of intestine discomfort following radiation therapy and can be alleviated by reducing the bowel radiation dose^27^.

In our study, aspiration pneumonitis, intestinal obstruction without a hernia, and mucositis were all linked to a longer duration of stay and higher mortality rates in patients undergoing radiotherapy. Given that RT disrupts mucosal barriers, resulting in mucosal alterations and muscle fibrosis, this is understandable. Aspiration pneumonitis is caused by a variety of factors, including acute and chronic radiation-induced mucosal changes, muscle fibrosis, and xerostomia. These factors often result in swallowing difficulties, increasing the risk of aspiration and aspiration pneumonia, as well as the duration of stay in the hospital^28–30^. Similarly, Berger et al. looked at the burden of oral mucositis across 65 studies, showed 30% patients required hospitalization due to mucositis^31^.

We also found that managing the complications of RT from these hospitalizations involved significant expenditures over a 10-year time period—$4.9 billion—leading to an annual estimated health-care burden of $408 million. This poses an enormous burden on healthcare resources. Surprisingly, there are very few studies that report the cost associated with treating the complications of RT at the national level^32^. The high hospitalization rate identified in our study could be used to identify patients whose treatment could be managed in an outpatient setting. Considering these findings, future studies should focus on estimating the cost associated with these complications across different healthcare settings, as well as different RT technology platforms, such as 2D, 3D, IMRT, proton, brachytherapy, etc. A better understanding of variables that predispose patients to toxicities leading to hospitalization could result in prospective interventional approaches. Recommendations for screening patients at high risk of complications in the outpatient setting and development of standardized management plans may have real world potential benefits to improve care and reduce hospitalizations.

## Limitations

E-codes were utilized to identify patients who had RT-related complications resulting in hospitalization. As NIS is an administrative database, E-code documentation which is not an obligatory requirement for reimbursement could result in underestimation of the actual hospitalization burden related to RT complications. Furthermore, the NIS does not contain data on tumor staging, concomitant medications, or the type and dose of RT administered, making it difficult to perform more sophisticated analysis. Furthermore, because NIS removes all personal identifiers for anonymity, successive readmissions of the same patient would be viewed as separate new admissions, leading to some overestimation. Despite these drawbacks, we chose NIS since other data sources for constructing a comprehensive picture of this subject are even more limited and less helpful in terms of national estimates. NIS is the largest all payer in-patient national database in the United States and its extensive reach and enormous size regarding data capture makes it more nationally relevant in comparison to other sources.

## Conclusions

RT-related hospitalizations are a small percentage of all hospitalizations. During 2005–2016, hospitalization rates for complications of RT remained almost same and did not show significant reductions; as a rough estimate, one in every thousand admissions to US hospitals occurs because of a RT-related complication, and this rate has remained unaltered between 2005 and 2016. The most common RT complication that required hospitalization were radiation cystitis, radiation-induced gastroenteritis and colitis, and esophagitis. When extrapolated to the national level for all admissions, the overall financial burden of managing RT related complications from these hospitalizations over a 10-year time period was $4.9 billion, leading to an annual estimated health-care burden of $408 million. Improved outpatient and emergency-room strategies for detecting and managing these complications could dramatically reduce the need for these hospitalizations. Identifying patients at greatest risk could also be useful as they could potentially be treated with more sophisticated techniques that reduce radiation dose to critical organs-at-risk, thereby likely diminishing toxicities and hospitalizations.

## Supplementary Information

Supplementary Information.

## Data Availability

The data that support the findings of this study are available from the corresponding author upon reasonable request.
