# Supplementary material for: Hospitalization rates from radiotherapy complications in the United States
Source: Sci Rep. 2022 Mar 14;12:4371. doi: 10.1038/s41598-022-08491-8 (PMC8921251; doi:10.1038/s41598-022-08491-8)
Supplement: Supplementary file 1 — Supplementary Information. [file 41598_2022_8491_MOESM1_ESM.docx]

**Supplemental Figure 1.** Flow diagram showing the inclusion criteria used for the study. Numbers in parenthesis are weighted estimates.


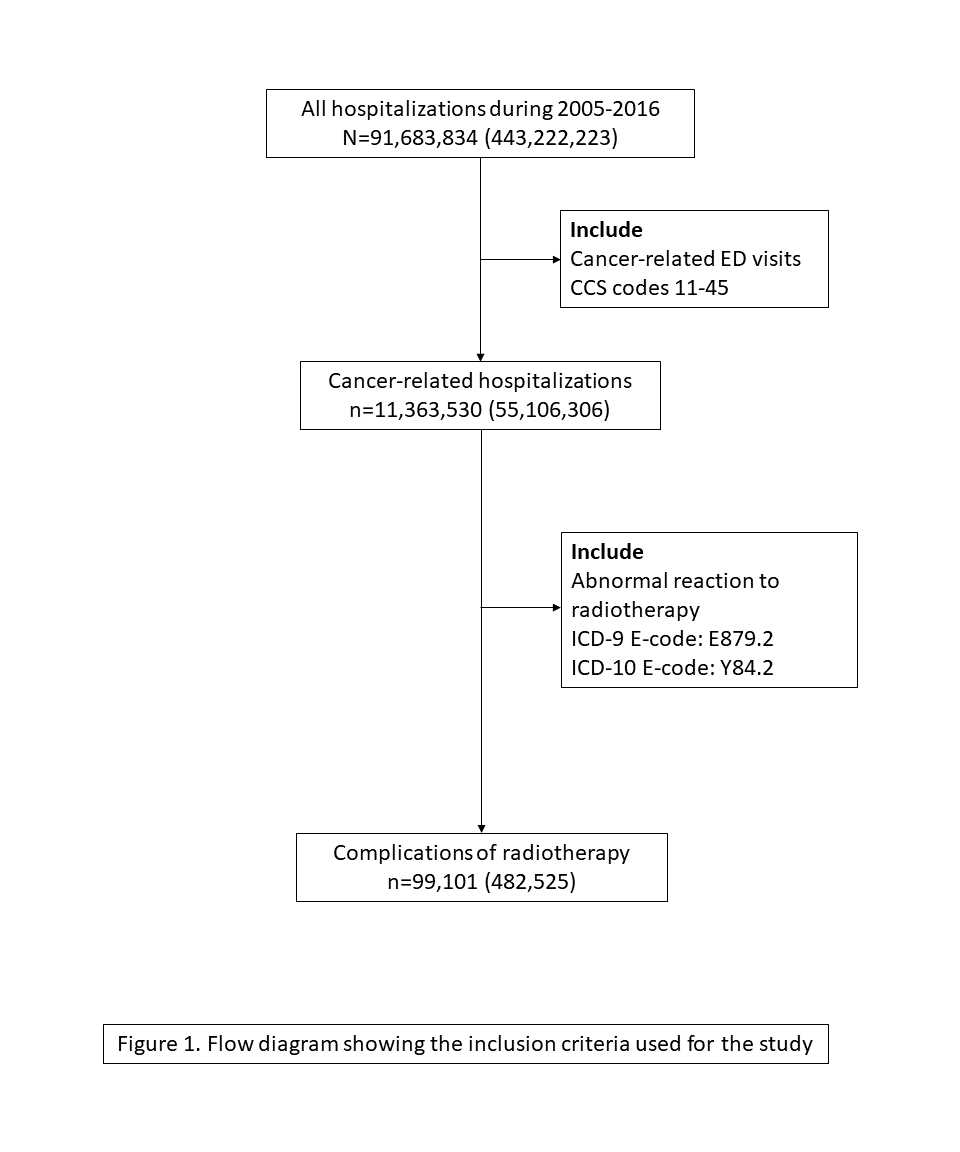


**Supplemental Table 1.** ICD and CCS codes used for defining complication of radiotherapy

| **Complications** | **ICD-9 Code** | **ICD-10 Code** | **CCS Code** |
| --- | --- | --- | --- |
| Intestinal obstruction (without hernia) | --- | --- | 145 |
| Radiation cystitis | 595.82 | N30.40 and N30.41 | --- |
| Radiation-induced gastroenteritis and colitis | 558.1 | K52.0 | --- |
| Disorders of rectum and anus | 569.4x | K62.6, K62.7, K62.8x, and K62.9 | --- |
| Esophagitis | 530.1x | K20.8, K20.9, and K21.0 | --- |
| Aspiration pneumonitis | --- | --- | 129 |
| Radiation-induced lung injury | 508.0 | J70.0 | --- |

**Supplemental Table 2.** Demographic characteristics of hospitalizations for complication of and radiotherapy

| **Variable** | **Complications of Radiotherapy** |
| --- | --- |
| Age |  |
| 0-17 | 6,913 (1.4%, 95% CI, 1.1%-1.7%) |
| 18-39 | 19,268 (4.0%, 95% CI, 3.5%-4.4%) |
| 40-64 | 183,752 (38.1%, 95% CI, 37.1-39.4) |
| ≥65 | 272,577 (56.5%, 95% CI, 55.1-57.2%) |
| Missing | 15 (0.0, 95% CI, 0.0%-0.0%) |
| Sex |  |
| Male | 254,971 (52.8%, 95% CI,51.1%-53.6%) |
| Female | 227,392 (47.1%, 95% CI, 45.9%-48.7%) |
| Missing | 162 (0.0%), 95% CI, 0.0%-0.0%) |
| Race |  |
| White | 340,360 (70.5%, 95% CI, 69.1%-71.2%) |
| Black | 47,375 (9.8%, 95% CI, 8.1%-10.7%) |
| Hispanic | 28,337 (5.8%, 95% CI, 4.3%-6.5%) |
| Asian or Pacific Islander | 9,414 (2.0%, 95% CI, 1.1%-2.7%) |
| Native American | 1,816 (0.3%, 95% CI, 0.0%-0.4%) |
| Other | 9,386 (1.9%, 95% CI, 0.7%-2.7%) |
| Missing | 45,838 (9.5%, 95% CI, 7.8%-10.6%) |
| Median household income |  |
| Quartile 1 | 114,936 (23.8%, 95% CI, 22.1%-24.8%) |
| Quartile 2 | 117,489 (24.3%, 95% CI, 23.1%-25.5%) |
| Quartile 3 | 120,469 (25.0%, 95% CI, 23.9%-26.6%) |
| Quartile 4 | 119,948 (24.9%, 95% CI, 23.3%-25.9%) |
| Missing | 9,682 (2.0%, 95% CI, 0.9%-2.8%) |
| Primary payer |  |
| Medicare | 279,274 (57.9%, 95% CI, 56.1%-58.9%) |
| Medicaid | 45,973 (9.5%, 95% CI, 8.3%-10.4%) |
| Private | 136,993 (28.4%, 95% CI, 17.3%-29.4%) |
| Self-pay | 7,895 (1.6%, 95% CI, 0.7%-2.5%) |
| No charge | 1,280 (0.27%, 95% CI, 0.11%0.37%) |
| Other | 10,414 (2.2%, 95% CI, 1.3%-3.5%) |
| Missing | 695 (0.14%, 95% CI, 0.0%-0.2%) |
| Region of hospital |  |
| Northeast | 100,508 (20.8%, 95% CI,19.1%-21.7%) |
| Midwest | 114,042 (23.6%, 95% CI, 22.1%-24.2%) |
| South | 178,712 (37.0%, 95% CI, 35.7%-38.2%) |
| West | 89,263 (18.5%, 95% CI, 17.8%-19.7%) |
| Bed size of hospital |  |
| Small | 55,370 (11.5%, 95% CI, 10.3%-12.7%) |
| Medium | 109,855 (22.8%, 95% CI, 21.3%-23.8%) |
| Large | 315,118 (65.3%, 95% CI, 64.3%-66.2%) |
| Missing | 2,181 (0.45%, 95% CI, 0.2%-0.5%) |
| Location/teaching status of hospital |  |
| Rural | 40,912 (8.5%, 95% CI, 7.3%-9.4%) |
| Urban nonteaching | 160,109 (33.2%, 95% CI, 32.1%-34.6%) |
| Urban teaching | 279,323 (57.9%, 95% CI, 56.1%-58.7%) |
| Missing | 2,181 (0.45%, 95% CI, 0.2%-0.6%) |
| Mortality | 17,226 (3.6%, 95% CI, 2.4%-4.6%) |
